# Supporting nurse practitioners’ practice in primary healthcare settings: a three-level qualitative model

**DOI:** 10.1186/s12913-017-2363-4

**Published:** 2017-06-26

**Authors:** Véronique Chouinard, Damien Contandriopoulos, Mélanie Perroux, Catherine Larouche

**Affiliations:** 1 University of Montreal Hospital Centre (CHUM), University of Montreal Public Health Research Institute (IRSPUM), C.P. 6128 succ. Centre-Ville, Montreal, Quebec, H3C 3J7 Canada; 2 University of Montreal, Faculty of Nursing, University of Montreal Public Health Research Institute (IRSPUM), Pavillon Marguerite-d’Youville, 2375, chemin de la Côte-Ste-Catherine, Quebec, H3T 1A8 Canada; 3 University of Montreal Public Health Research Institute (IRSPUM), C.P. 6128 succ. Centre-Ville, Montreal, Quebec, H3C 3J7 Canada

**Keywords:** Primary healthcare nurse practitioners, Primary healthcare service organization, Scope of practice, Support practices

## Abstract

**Background:**

While greater reliance on nurse practitioners in primary healthcare settings can improve service efficiency and accessibility, their integration is not straightforward, challenging existing role definitions of both registered nurses and physicians. Developing adequate support practices is therefore essential in primary healthcare nurse practitioners’ integration. This study’s main objective is to examine different structures and mechanisms put in place to support the development of primary healthcare nurse practitioner’s practice in different healthcare settings, and develop a practical model for identifying and planning adequate support practices.

**Methods:**

This study is part of a larger multicentre study on primary healthcare nurse practitioners in the province of Quebec, Canada. It focuses on three healthcare settings into which one or more primary healthcare nurse practitioners have been integrated. Case studies have been selected to cover a maximum of variations in terms of location, organizational setting, and stages of primary healthcare nurse practitioner integration. Findings are based on the analysis of available documentation in each primary healthcare setting and on semi-structured interviews with key actors in each clinical team. Data were analyzed following thematic and cross-sectional analysis approaches.

**Results:**

This article identifies three types of support practices: clinical, team, and systemic. This three-level analysis demonstrates that, on the ground, primary healthcare nurse practitioner integration is essentially a team-based, multilevel endeavour. Despite the existence of a provincial implementation plan, the three settings adopted very different implementation structures and practices, and different actors were involved at each of the three levels. The results also indicated that nursing departments played a decisive role at all three levels.

**Conclusions:**

Based on these findings, we suggest that support practices should be adapted to each organization’s environment and experience and be modified as needed throughout the integration process. We also stress the importance of combining this approach with a strong coordination mechanism involving managers who have in-depth understanding of nursing professional roles and scopes of practice. Making primary healthcare nurse practitioner integration frameworks more flexible and clarifying and strengthening the role of senior nursing managers could be the key to successful integration.

**Electronic supplementary material:**

The online version of this article (doi:10.1186/s12913-017-2363-4) contains supplementary material, which is available to authorized users.

## Background

Several studies have shown that greater reliance on nurse practitioners can alleviate pressures on primary healthcare systems, such as those caused by public expenditure cuts and demographic changes. This cost-effective measure has the potential to improve primary care services accessibility without lowering quality of care or patient satisfaction levels [1–8].

Their integration into primary care is, however, not devoid of challenges, notably because the role attributed to primary healthcare nurse practitioners (PHCNPs) expands nursing practice and consequently implies rethinking the nature of each professional’s role in primary healthcare teams [9]. Developing adequate team-focused support practices is thus essential for PHCNPs’ integration and ultimately for improving those teams’ capacity to contribute to primary healthcare quality accessibility and efficiency [10]. We define support practices as all activities intended to respond to the needs of the PHNCP or of other professionals collaborating with the PHNCP, and analyze them from a multi-level perspective – clinical, team, and systemic.

While there is relatively abundant literature on the advantages and challenges of integrating nurse practitioners into primary care settings, the optimal structures and practices to support their integration have remained largely unexplored [10, 11].

The present study focuses on three primary care settings in Quebec (Canada) to examine the types of managerial structures and mechanisms put in place to support the development of PHCNPs’ practice. We suggest dividing support practices into three types—clinical, team and systemic—for a thorough understanding of their impact on PHCNPs’ integration. Our analysis of these three types of support demonstrates that practices should be adapted to the environment and experience of the organizations and be modified as needed throughout the integration process. In a broader perspective, our results also indicate that nursing departments play a decisive role in PHCNPs’ integration at all three levels. We suggest that clarifying and strengthening their role could therefore be the key to successful integration.

### The challenges of integration

PHCNPs practising in the province of Quebec are registered nurses who have successfully completed a university master’s-level nurse practitioner program. According to Quebec’s 2010–2015 strategic health plan, PHCNPs are expected to provide primary care in three practice environments: local community health centres (CLSCs—public organizations providing primary care and social services); hospital-based family medicine units (FMUs—hospital affiliated medical clinics which train medical residents in family medicine); and family medicine groups (FMGs—private medical clinics where public hospitals cover the salary and benefits of nursing staff in exchange for clinics’ providing extended opening hours and increased care continuity) [10]. Regardless of their practice environment, it is mandatory for every PHCNP in Quebec to sign a “partnership contract” with one or more family physicians stipulating rules of collaboration as well as each one’s professional roles and responsibilities.

According to Quebec’s regulatory frameworks, PHCNPs technically come under the responsibility of a nursing department. These nursing departments are present in every Health and Social Services Centre (CSSS),^1^ and are generally responsible for setting and pursuing the vision for high-quality nursing services and ensuring the conditions are in place to achieve this quality in the various organizations making up the CSSS. However, on the ground, the responsibility for PHCNPs’ integration often involves many other actors apart from the nursing departments.

The scientific literature and various reports have shown that, at the local level, a number of obstacles can hinder successful insertion of PHCNPs into primary healthcare settings, such as feelings of isolation in mainly medical-centred environments, limited opportunities to communicate with other nurses from similar settings, lack of team preparation prior to PHCNPs’ integration, confusion in role definitions, and misuse of the nurses’ capacities [12–14]. Poghosyan et al.’s quantitative study, in which nearly 600 PHCNPs were surveyed, indicated that they were often dissatisfied with their relationship with their immediate supervisor [15]. Certain features of many healthcare systems (i.e., wide variety of practice settings, physical distance between key players, differences in legal frameworks) also add to the difficulty of successfully developing this advanced practice.

### A three-level approach to support practices

The scientific literature frequently highlights the importance of support practices to overcome the main integration challenges presented above [11, 16–22]. However, it rarely proposes specific practical models for planning and implementing adequate support practices [10, 11], most likely because support practices encompass a wide array of activities and the meaning of the concept remains vague.

We define support practices as all activities that are intended to respond to the needs of the PHNCP or of other professionals collaborating with the PHNCP. Support practices are designed to resolve problems, meet challenges, or improve certain processes [23]. They can take various forms (e.g. clinical, psychological, administrative) and be implemented at different organizational levels [24, 25]. To capture these organizational levels of support practices—going from the local to a broader macro level—we organized practices into three categories: clinical, team, and systemic. These three forms of support overlap in many ways, and clearly distinguishing among them is sometimes difficult when observing actual practices on the ground. However, since healthcare organizations are complex systems in which various structures and processes interact at different levels, disaggregating the concept of support into its multiple components is useful to identify which support practices are more effective. The next sections provide more detail on the definition and delimitation of the multi-level support practices model we developed and used for this study.

#### Clinical support

What we defined as ‘clinical level’ support consists of interventions aimed at facilitating PHCNPs’ clinical work. It includes the most immediate aspects of support in their work environment, such as access to clinical information and resources, capacity development opportunities, and training, as well as measures to help them occupy the full scope of nursing practice. For example, relationships between PHCNPs and their partner family physicians [16, 20], and opportunities to exchange knowledge and experience with other nurse practitioners or clinical nurses occupying similar roles in other settings, are clinical support elements that affect PHCNPs’ work and capacity development [26, 27]. MacPhee [26] argues, along these lines, that nurse managers are indispensable collaborators in transferring information and resources to PHCNPs.

#### Team support

A PHCNP’s arrival in a new practice environment requires mutual adaptation, as the PHCNP adjusts to the team’s established patterns of functioning, while the team adjusts its functioning to include the PHCNP. ‘Team level’ support consists of measures taken to reorganize roles, redesign task distribution, and manage interpersonal relations in a team before and after the PHCNP’s integration. Among the few studies on this type of support, those that have focused on challenges related to managing relationships and redesigning roles within the team suggest that fostering role complementarity, increasing communication channels to reduce hierarchical differences, and building common goals while leaving space for team members to organize themselves and manage their own difficulties are keys to successfully meeting those challenges [19, 28]. Reay et al. suggest adopting a ‘balcony’ perspective, from which the manager focuses not only on the nurse practitioners, but on the team as a whole, and formulates overall goals to guide members in their actions and adaptation to changes [19]. In all cases, integrating a new role into a team can be seen as an opportunity to rethink the team’s organizational structure [9, 29].

#### Systemic support

‘Systemic level’ support practices are those related to adaptations *to* and *of* the broader environment within which PHCNPs are integrated. Cameron and Masterson [30] argue that nurse managers can play a crucial role in nurse practitioners’ integration into primary care settings, but that their capacity to do so depends on their level of knowledge regarding this new role, on their capacity for strategic action within the organization, and on the level of responsibility attributed to them in this function. Other systemic support practices pertain to financial support or service reimbursement rules [11, 22, 31] and the constitution and organization of coordinating committees [11]. Notably, studies on the topic highlight the importance of including nurse managers in regional coordinating committees related to PHCNPs and of establishing good communication mechanisms between PHCNPs and nursing departments [11, 32].

## Methods

This study was part of a larger multicentre study focused on formulating recommendations regarding PHCNPs’ integration and best practices in Quebec’s healthcare system [9, 10]. The project was based on six case studies of settings deemed by Quebec’s Ministry of Health and Social Services (MSSS) to be successful examples of PHCNPs integration. Each case is defined as a clinical team into which one or more PHCNPs have been integrated. Beyond sharing the characteristic of being perceived as successful examples, cases were selected according to a logic of maximum of variation sampling in terms of location, organizational setting, and stages of PHCNP integration.

Findings in the present article focus on three of the above six cases. They are based on the analysis of available documentation in each setting and on 18 semi-structured interviews with key actors in each clinical team: PHCNPs, physician partners of the PHCNPs, directors of nursing in the CSSSs, administrative staff, and other nurses. Interviews were conducted in the healthcare settings and lasted about an hour each.

Interviews were audio-recorded, transcribed, and read for accuracy. Interviews were conducted in French (citations in this article have been translated into English). All data were compiled and coded following a thematic analysis approach (systematic identification of recurring themes) [33, 34]. We employed two methods to interpret the data. First, we used pattern matching to highlight patterns linking data from the literature with empirical data and patterns appearing within the interviews [35]. The primary structure used to organize and code data was the typology of support levels presented above. We then performed cross-sectional analysis by comparing similarities and differences between the case studies.

## Results

The three cases selected for in-depth analysis were a small CLSC in a rural area, a small FMG in a rural area, and a large FMU in an urban setting. Stage of NP integration ranged from two to 7 years, and the three settings had significantly different organizational structures. In the first case, PHCNPs were under the direct responsibility of the CSSS Director of Nursing, while in the two other cases, supervision was shared between at least two players. PHCNPs in the second setting reported structurally to the Assistant Director of the General Services Department and functionally to the Director of Nursing, whereas PHCNPs in the third setting reported to an administrative assistant while receiving clinical advice from the CSSS Director of Nursing and a nursing practice clinical manager (see Table 1). Lastly, patient management in the settings under study followed either a ‘joint’ or a ‘consultative’ model. A model is considered joint when the NP and the physician partner follow the same panel of patients. In such a model, both professionals may see the same patients at different points in their treatment. Conversely, a model is considered consultative when the NP and the physician partner each follow a different panel of patients and the physician is consulted as needed. [10, 36, 37].

I. Clinical support

**Table 1:**
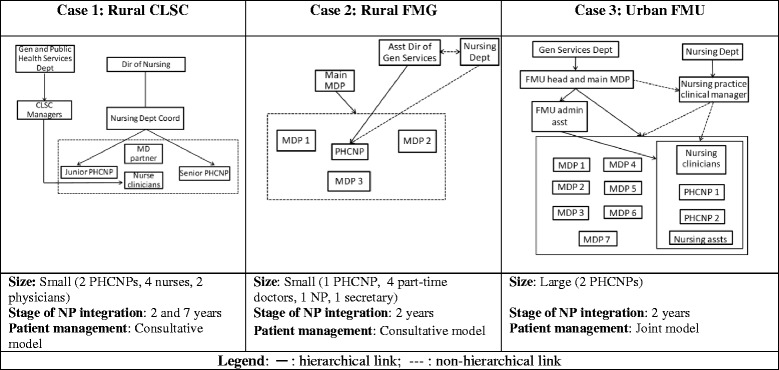
Case description and management structures

Analysis of the three cases showed, first, that the nursing department always took part in clinical support, regardless of which department was officially supervising the PHCNPs. However, direct supervision by the nursing department, as in Case 1, seemed to translate into higher levels of support. In the two other cases, PHCNPs had to deal with two different managers and a complex hierarchical structure even though, in practice, they often requested the help of nursing department managers before consulting their immediate supervisor. More than direct clinical support however, the vertical support offered by the nursing departments was mostly focused on facilitating exchanges among PHCNPs. Coordinators from the nursing department would, for example, organize exchanges among PHCNPs working in different settings on a regular basis. The coordinators used these occasions to stay in regular contact with PHCNPs despite geographical distance, identify their training needs, and become informed of potential difficulties arising in each setting. All participants in the study referred to this as an invaluable form of support uniting managers and clinicians.

Within the healthcare setting, horizontal support between PHCNPs emerged as another important aspect of clinical support. Such exchanges generally consisted of sharing clinical experiences, documentation, and advice on day-to-day work arrangements in the clinic. Even in cases where no formal measures were taken to support horizontal exchanges (Case 2), the PHCNP maintained contacts with other PHCNPs in more or less informal ways, such as meeting outside work hours. The existence of this parallel network shows the need for this type of support. Another successful horizontal support practice was the dyadic integration of nurses in the healthcare settings. Although the relationship between the two nurses varied according to their years of experience (senior–junior in Case 1 and junior–junior in Case 3), the fact of there being two PHCNPs in one setting gave them the opportunity to create alliances, develop and share a vision of their role, validate ideas related to their clinical practice or their integration into the setting, and suggest, when needed, changes to make the most of advanced nursing practices.

“It was a very important source of support. It was so stressful in the beginning. It’s like another way to work, everything is new. So, with two of us, I was able to compare what I was going through with someone…that, for me, I felt like this, like that…” (PHCNP, Case 3).

Being at least two in the same clinic also presented advantages in terms of work efficiency and coverage during absences.

Our analysis also showed that patient management was a challenge when more than three partner physicians were involved, especially in settings based on a joint patient management model. The consultative model (Cases 1 and 2) was easier to manage and fostered collaborative relationships between MDs and PHCNPs, whereas the joint model (Case 3) tended to lead more toward supervision-based relationships. Apart from the question of the number of physicians, most of the coordination mechanisms between physicians and PHCNPs seemed to be based on mutual adjustment and were modified over time. In all cases, the need for physician support was much higher in the first 6 months and diminished as the PHCNP developed greater confidence and expertise. As the PHCNP developed complementary services (Case 2), relationships with the physician became increasingly consultative and less interdependent. Certain formal measures, such as narrowing the type of caseload selected (Cases 2–3) and having a set consultation plan between PHCNP and physician (Case 2) helped PHCNPs develop independence and fostered satisfying interprofessional consultations. The need for formal mechanisms depended mostly on setting size and quality of communication between members.

II. Team support

There was no uniform process common to all three cases to support the teams after the introduction of the PHCNPs. Team support was provided by different institutions and people inside and outside the team itself. In the smaller settings (Cases 1 and 2), integration processes seemed to work more smoothly, as logistical adjustments were simpler than in the larger setting and communication between members of the smaller teams was generally more fluid.

Nursing managers seemed to be the most useful source of support for the PHCNPs, as they could help in defining and developing the PHCNPs’ role and provided a broader vision of the whole process. The general services managers (immediate supervisors in Cases 2 and 3), on the other hand, did not have extensive knowledge of the PHCNPs’ role and so their support was limited to basic financial and administrative interventions (schedule planning, space assignments, etc.).

The physicians in charge of the clinics also appeared to play an important role in team support. They were invited to participate in evaluating the work of PHCNPs coordinated by the nurse managers. They also organized meetings to facilitate communication between all professionals in the clinic and served as a reference point for questions and comments from other physician partners on the PHCNP’s role. They were more invested in dealing with day-to-day relationships among team members, while the nursing department managers adopted more of a counselling role. In general, the head physicians had, to a certain extent, developed their own mechanisms for supervising and integrating the PHCNPs into their clinics.

Thus, although nursing department managers, physician managers, and the PHCNPs’ partner physicians shared many responsibilities for PHCNPs’ integration, they seemed nevertheless to operate in relatively parallel networks, with few structures facilitating communication between them. In some settings, notably in Case 2, where the physicians did not come under the financial and administrative responsibility of the CSSS, the nursing department managers felt they did not have the necessary legitimacy to intervene in the healthcare setting, even though they were officially in charge of the PHCNPs’ integration and of paying their salary. They had to rely essentially on the personal collaboration links they had established with the clinic’s head physician to support, indirectly, the PHNCPs.

“We can encourage the physician in charge of the clinic to think about this. We can do that, but we can’t necessarily force him to say… ‘look, us, we’d like our nurse practitioner to do such and such.’ It’s always a matter of collaborating with our physician in charge.” (Assistant to the General Services Department Director, Case 2).

III. Systemic support

With regard to systemic support, the nursing department was again the key player. However, while responsibilities for clinical and team support were generally delegated to a nursing department manager, it was the Directors of Nursing (a position often labeled as Chief Nursing Officer in other jurisdictions) themselves who took on most of the systemic, political, and macro-level tasks pertaining to the recognition of PHCNPs’ practices. One example mentioned a few times was the fact that the nursing directors represented the PHCNPs’ interests at regional and provincial levels regarding recognition of their right to prescribe diagnostic tests or drugs. These types of interventions occurred at all stages of PHCNPs’ integration in primary care delivery structures. The nursing department was also involved in advocacy and education to promote the administrative integration of additional PHCNPs in other institutions within the CSSS. Despite their involvement in such support activities, the Directors of Nursing had few formal opportunities to meet with colleagues from other CSSSs in their region, and some had built their own informal networks with other nursing departments.

According to the integration plan developed by the MSSS, systemic integration was to be supported by local and regional implementation committees. However, while our respondents saw the role of the nursing department as unequivocally central in systemic support practices, their opinions about the role and usefulness of these committees were generally mixed. Only one primary healthcare setting had established an official local implementation committee, and the three settings had variable experiences with the government-mandated regional committee. In general, the need for such structures did not seem very pressing after the first stages of PHCNPs’ integration, since these committees were intended to plan broad general directions rather than to discuss emerging problems. As the actors were already in regular interaction with each other in the CSSS, and most decisions were taken outside of formal meetings, this additional committee was seen as not really necessary.

## Discussion and recommendations for practice

A first observation to be made is that there does not seem to be a unique and straightforward process to integrate PHCNPs and support development of their practice in primary care settings. The three settings studied were examples of relatively successful integration, but all relied on very different implementation structures and practices. Our interpretation is that each setting had to invent its own support model from resources available in its organization, past experience, and managers’ personal interests, strengths, and weaknesses. Although the government’s implementation plan included structural elements, those were poorly suited to local practices and lacked support from participants.

The major drawback of this high variability in local practices was that most issues were addressed in an ad hoc way, leading to overlaps and the involvement of many actors from different organizational levels. This was especially true in larger settings, as it was more difficult to coordinate changes and adjustments on an informal basis, given the complexity of the structure and the large number of team members. The absence of standardized management structures and practices was less of a challenge in smaller contexts, where there was greater reliance on individual and informal coordination mechanisms. It should also be mentioned that, in all cases, despite the existence of formal coordination structures, many of the communication processes between PHCNPs, nursing departments, and the settings were, in practice, based on informal and personal relationships.

The contingency theory approach developed in the field of organization studies can be helpful in formalizing those observations. More specifically, from a contingency theory perspective, size matters. Structural arrangements that make sense in a large organization might make no sense at all in a small one [38, 39]. The same logic applies to other parameters, such as environment complexity. Implementation processes whose evolution is difficult to predict and that are carried out in complex environments (diffuse responsibilities and participation) are ill-suited for standardized structures. For example, regarding PHCNPs’ implementation in primary care, it might make more sense for the MSSS to simply list best practices and set targets and schedules rather than to establish committees and appoint participants. Moreover, any implementation process is, by its very nature, evolving. Thus implementation structures and practice will have to adapt and evolve as well. The gradual transformation of the main physician partner/PHCNP clinical relationship from strict supervision to counselling and collaboration is one example of adaptation to changing needs [27]. Ultimately, the goal should be to achieve a balance between a shared and broad vision of the PHCNP’s role, on one hand, and the constraints and needs of the clinical environment, on the other [40].

Even though one set of support structures and practices cannot benefit and be relevant to all settings equally, two general recommendations can still be formed in light of the successful support practices we observed in this study. The first relates to the role of the nursing department. Despite the different responsibilities attributed to the nursing departments in each case study, in all cases, and at all three levels of support, the Director of Nursing or a representative had a central and positive effect on the integration process; hence the importance of clarifying and supporting the nursing department’s responsibilities. In contrast to the General Services Department, which usually offered only basic administrative support, the nursing department’s interventions also had the objective of developing a broader vision of what PHCNPs’ role in primary care should be. Supporting and clarifying that department’s responsibilities would also be helpful to reduce the number of actors involved and the overlapping of tasks. Our findings on the centrality of the role played by the nursing department in integrating PHCNPs in primary care institutions are consistent with those of other studies on the topic [11, 26, 30, 32]. The three levels of support approach, however, constitutes an additional tool to clarify the role of nursing departments and understand how it can be most productively enacted at each support level. Notably, our findings showed that the nursing department’s role was not limited to the clinical level, but also contributed significantly at the team and systemic support levels. This strongly advocates, on one hand, for assigning nursing departments the responsibility for PHCNPs’ direct supervision [41] and, on the other, for giving nursing departments the means to be informed and included in team and macro level support structures and practices right from the beginning of the integration process, even if they do not have direct authority in certain primary care settings. It appears there is still room for improvement in that area, given the lack of legitimacy felt by the nursing department managers when intervening at the team level, for example.

The second recommendation also relates to the role of nursing actors in support practices but, in this case, at the horizontal level. For example, the PHCNPs all stressed the positive impact of there being more than one PHCNP in the same clinic, as it provided opportunities for exchange and communication. Fostering horizontal support would also make it possible to take advantage of senior PHCNPs’ experience to train junior PHCNPs, thereby reducing reliance on physicians for such training. Moreover, in contexts where the number of nurse practitioners being integrated into primary care settings is meant to increase, being able to share successes and challenges with those who have already experienced the process becomes even more vital. At a higher level, nursing administrators and managers also benefit from horizontal exchanges with other nursing departments in the region. Formalizing the time commitment and creating meeting opportunities could be useful to support this need for horizontal support.

## Conclusions

Our analysis suggests that PHCNPs’ integration and continuing development in primary care settings are more than a local matter. This tallies with other results in the field suggesting that PHCNPs’ integration is not a straightforward process. Because PHCNPs’ scope of practice and roles are at the intersection of medicine and nursing, their integration challenges existing role definitions of both registered nurses and physicians. As other studies have found [11, 17, 21, 31, 36, 41], managers trained in nursing seem better equipped than their non-nursing counterparts to conceptualize the integration process in terms of scope of practice and role redefinition, and this appears to be more productive than working from a narrower task-based perspective. However, this general observation needs to be considered in the light of specific contexts. For example, in our study, PHCNPs’ partner physicians also played a central role, as they were the ones best positioned to support PHCNPs’ clinical role development.

Our study makes two original contributions to the field. The first is in suggesting that supporting PHCNPs’ integration is a multilevel endeavour. As we have argued, there are distinct needs and responsibilities at the clinical, team, and systemic levels. Some actors may be involved at all three levels, but it is likely that different actors will be better positioned for different levels of support, making PHCNPs’ integration an intrinsically team-based process. This brings us to the second contribution to the field, which is the observation that, as support practices for PHCNP integration involve a variety of actors from different backgrounds and structural positions, there is a need for strong but adaptive coordination structures. A balance must be struck between relying on highly formalized statutory committees—which will likely be less adaptable—and counting exclusively on informal communication and personal involvement, whose effectiveness is unpredictable and unreliable. This is where contingency theory arguments—that there are no best organizational structures to produce strong but adaptive coordination—provide a useful conceptual framework. The best structure will be the one best fitted to the contingencies of a given organizational system. When designing the structure for PHCNPs’ integration support, it is essential to leave enough room for local adaptation while making sure there are people accountable for the outcomes. This goes back to the importance of having a senior manager—someone with in-depth understanding of professional roles and scopes of practice—coordinating the functioning of implementation structures from a ‘balcony’ or ‘whole-picture’ vantage point [19].

## Additional file

Additional file 1: Interview Guide (Combined). (DOCX 34 kb)

## Footnotes

1 At the time of the study, Health and Social Services Centres (*Centres de santé et de services sociaux*) were mandated to oversee the activities of public health institutions on a given territory, including CLSCs, rehabilitation centres, long-term care facilities, and often hospitals. The system has since been modified (in 2015), resulting in further centralization and administrative integration of institutions into what are now called CISSS: *Centres intégrés de santé et de services sociaux*, and CIUSSS: *Centres intégrés universitaires de santé et de services sociaux*.
