# Supplementary material for: Supporting nurse practitioners’ practice in primary healthcare settings: a three-level qualitative model
Source: BMC Health Serv Res. 2017 Jun 26;17:437. doi: 10.1186/s12913-017-2363-4 (PMC5485609; doi:10.1186/s12913-017-2363-4)
Supplement: Additional file 1: — Interview Guide (Combined). (DOCX 34 kb) [file 12913_2017_2363_MOESM1_ESM.docx]

Interview Guide (Combined)

# Prior to the interview:

## Sending the consent form

## Presenting the objectives of the current interview

## Presenting the use of the material collected and the procedures for protection of confidentiality

## Signing the consent form

# Description of the setting:

## Could you describe your CSSS/clinic in a general way?

### Workforce/types of professionals

### Clientele (socioeconomic, geographic)

# Training/experience

## Could your briefly describe to me your training as well as your work experience?

## How long have you been working here?

# Introduction of PHCNPs in a practice setting

## How did the idea of having PHCNPs within your CSSS/clinic first come up?

## Did you participate in any team discussions where the idea was presented or debated?

## If yes, follow up to obtain further details on:

### The moment when these discussions took place (at the beginning or end of the decision-making process)

### The persons present during the discussions

### The main issues raised?

### Any controversial/contentious aspects

## According to you, did the local committee for implementing PHCNPs within CSSS or the regional committee at the regional public health department level (ASSS) play a role in the decision-making process that led to setting up PHCNPs within this clinic? If yes, what role?

*(For PHCNPs only:)*

## Are you aware of what was done before the start of operations to prepare for your arrival?

### Involvement of local and regional committees

### Role of the Director of Nursing (DN)

### Involvement of physicians

# Preparation of the environment:

*(For DNs, managers, and physicians: )*

## Can you describe to me how the arrival of PHCNPs took place?

## Where any steps/actions taken to prepare for the arrival of PHCNPs?

## Were other actors mobilized for the introduction process? If so, which ones, and what was their involvement?

## Follow-up to find out whether the elements below were addressed before PHCNPs began functioning.

### Necessary physical spaces

### Setting salaries/salary grids

### Discussion or training sessions on PHCNP roles and practice

## What were the main challenges that you encountered while preparing for the arrival of PHCNPs?

*(For physicians, nurse clinicians, and other professionals: )*

## Were you involved in the steps to prepare for the arrival of PHCNPs?

## Was the arrival of a PHCNP a topic that came up in informal discussions with your colleagues?

## Was the DN of the CSSS, or someone representing her, involved in discussions concerning the introduction of the PHCNP?

*(For PHCNPs: )*

## Could you describe to me how your arrival at the clinic went?

### Meetings with other professionals

### Role of the partner physician in receiving you

### Role of other clinicians/administrators in receiving you

### Information provided to different partners on the nature and form of PHCNP practice

## During the introduction process, how did your orientation period go?

# Implementation of the role

*(For DNs and managers: )*

## What support and monitoring activities and mechanisms for PHCNP practice have you used or put in place?

## What are the main challenges you face in supporting PHCNP practice?

## Have any PHCNPs shared with you any concerns regarding working conditions thus far?

*(For DNs, managers, and physicians: )*

## Have PHCNPs already contacted you to discuss challenges related to the implementation of their role? If so, what were their concerns?

*(For PHCNPs: )*

## Could you describe a typical work day at the clinic to me?

## How do you think that others perceive your role?

(*For physicians, PHCNPs,* *nurse clinicians, and other professionals)*

## According to you, what is the role of a family physician? / of a PHCNP? / of a nurse clinician in an FMG?

## How did you go about setting up the sharing of responsibilities between the different professionals?

### Who was involved?

### What motivated your choices?

## How do you view the DN’s role with respect to the definition of roles?

*(For all)*

## Are there any formal meetings/mechanisms within the establishment for follow up with PHCNPs? Or does it rather happen through informal discussions?

# Division of work/responsibilities

*(For physicians, nurse clinicians, and other professionals: )*

## In your daily work, what are your interactions with the PHCNP?

## Do you see benefits to working in a team that includes a PHCNP?

## Do you see disadvantages to working in a team that includes a PHCNP?

## Since the PHCNP has been deployed, have you noticed any changes in the way the clinical team functions?

*(For nurse clinicians and other professionals: )*

## Did the introduction of a PHCNP had any kind of effect on your work?

## When one of your patients needs a diagnosis, treatment, or test, how do you decide whether to refer to the PHCNP or to a physician?

*(For PHCNPs and physicians: )*

## How are decisions made to determine who sees which patients?

## When a decision has to be taken concerning a patient:

### How does it happen?

### Who decides?

### How is responsibility assigned?

## Did any adjustments have to be made to allow the PHCNP to exercise her authority to prescribe (Dx and Rx)? If so, how and by whom were these adjustments negotiated? Were you involved?

# Interprofessional collaboration

*(For DNs and managers: )*

## Did the introduction of PHCNPs lead to changes in your organization?

## Since PHCNPs have been deployed, have you noticed any changes in the way that clinical teams function?

## Do you have the impression that the role of the PHCNP is well understood by all of the teams?

*(For physicians, PHCNPs, nurse clinicians, and other professionals: )*

## When there is a disagreement between you and another professional, how is the disagreement managed?

(For all)

## Are there any mechanisms set up by the clinic to maintain, or even improve, collaboration? If so, could you tell me about them?

# Conclusions

*(For physicians: )*

## In hindsight, what expectations/misgivings did you have regarding the introduction of PHCNPs in your work setting?

*(For nurse clinicians and other professionals: )*

## Compared to what you imagined before the arrival of an PHCNP, are there things that have surprised you in terms of what collaboration means in such a context?

*(For DNs, managers, physicians, and PHCNPs: )*

## Based on the experience you have gained, do you have suggestions on how the introduction of a PHCNP should be organized?

### Basic elements to be clarified from the start

### Introduction process

### Conditions for success

*(For all: )*

## Are there any elements in the integration process or in the development of high-performing clinical practice modes that you feel are still problematic?

# General conclusion

## Conclude by discussing potential collaboration on issues left outstanding

## Thanks and presentation of the next steps
